# Advances in plant phenomics: From data and algorithms to biological insights

**DOI:** 10.1002/aps3.11386

**Published:** 2020-09-01

**Authors:** Sunil K. Kenchanmane Raju, Addie M. Thompson, James C. Schnable

**Affiliations:** ^1^ Department of Plant Biology Michigan State University East Lansing Michigan 48824 USA; ^2^ Department of Plant, Soil, and Microbial Sciences Michigan State University East Lansing Michigan 48824 USA; ^3^ Michigan State University AgBioResearch East Lansing Michigan 48824 USA; ^4^ Center for Plant Science Innovations University of Nebraska–Lincoln Lincoln Nebraska 68583 USA; ^5^ Department of Agronomy and Horticulture University of Nebraska–Lincoln Lincoln Nebraska 68583 USA

The measurement of the characteristics of living organisms is referred to as phenotyping (Singh et al., 2016). While the use of phenotyping in plant biology and genetics can be traced back at least to Gregor Mendel sorting and counting peas by shape and pod color 160 years ago, addressing current questions in plant biology, genetics, and breeding often requires increasingly precise phenotyping of a wide range of traits. Accurate phenotyping has played a role in both novel discoveries about the fundamental biology of plants and the development of improved crop varieties around the world. With the advent of inexpensive genotyping tools, crop functional genomics has entered the “big data” era, but efficient large‐scale phenotyping is still an impediment hindering plant functional genomics. The precise measurement of plant traits both throughout the growth cycle and across environments is expensive and labor intensive. A convergence of interdisciplinary efforts has led to the development of new technologies for nondestructive phenotyping in plants to measure large numbers of traits accurately with higher throughput (Close and Last, 2011). Improvements in imaging and automation, as well as in data processing and analytics, are helping to fill significant gaps in efforts to employ these new technologies to connect genetic variation with phenotypes (Yang et al., 2020). In recent years, plant phenomics research has transitioned from the development of methods and molecular genetic analysis of model plants in controlled environments toward accelerated efforts for applications in plant breeding, association studies, and stress phenotyping in crops grown under complex field conditions (Costa et al., 2018). In this special issue, “Advances in Plant Phenomics: From Data and Algorithms to Biological Insights,” we present six papers that capture plant phenomics extending to multiple scales, from field‐wide traits, to individual plots or plants, to specific gene interactions.

In the context of field‐scale image acquisition and processing, one of the first challenges that must be addressed in drone‐based imaging of agricultural fields is turning free‐flown images acquired over an area into a single mosaic image from which phenotypes can be extracted. Current methods rely mostly on the ability to locate each pixel in space, requiring costly global positioning systems (GPS) and/or inertial measurement units (IMU) to track the position of ground control points relative to the image acquisition device. These approaches are computationally taxing, demand larger data storage, and require the purchase of software licenses, leading to a high barrier of entry. Aktar et al. (2020) have developed a method called Video Mosaicking and summariZation (VMZ) to provide an alternative pipeline that is faster, less computationally demanding, and much cheaper to implement. The authors show that compared to other methods, VMZ not only works faster but also produces mosaics with superior quality. This work, demonstrated here in maize, begins to democratize drone‐based phenotyping for large‐ and small‐scale field researchers across multiple species.

Although many field‐based plant phenotypes can be aerially captured, proximal sensing techniques can also connect physical phenomena with biology. Resistance to lodging is an essential aspect of plant standability, affecting grain yield and quality. While susceptibility to lodging is known to have a genetic component, differences in the timing and severity of damaging winds and rainfall can make it challenging to quantify lodging consistently from year to year. Hence, despite the prevalence and detrimental economic impact of lodging, there is a lack of adequate protocols for assessing lodging and connecting field‐based mechanical methods to the underlying plant biology. Erndwein et al. (2020) summarize field‐based mechanical phenotyping methods currently used to assess lodging resistance in cereal crops. The authors discuss the complexity of accurately phenotyping natural lodging failures. By also focusing on devices used to measure root lodging in cereals, this review fills the gap and complements the well‐studied aspects of stalk lodging, biomechanics, and lab‐based mechanical phenotyping devices (reviewed in Berry et al., 2004; Niklas and Spatz, 2012; Shah et al., 2017). The authors provide an overview of the current status of field‐based phenotypic approaches, discuss possible reasons for the lack of reproducibility between studies and between devices, and provide suggestions for best practices. This review is accessible to those without a background in biomechanics and enables them to use the right tools or method for measuring lodging resistance in breeding programs, which is necessary to connect field‐based mechanical measurements with the underlying genetic architecture of lodging for crop improvement.

While environmental factors can radically reshape the canopy through events such as lodging, more subtle variations in plant canopy architecture play a substantial role in the efficiency of light capture and utilization, as well as water use efficiency (Peng et al., 2020). Of the many traits that contribute to overall canopy architecture, leaf angle has been the focus of particular genetic investigation as it has been linked to increased grain yield (Duvick, 2005). However, the manual measurement of leaf angle is labor intensive and relatively low throughput and therefore expensive when large numbers of data points are required. Kenchanmane Raju et al. (2020) report the development of a semi‐automated, MATLAB‐based software framework, Leaf Angle eXtractor (LAX), for the estimation of leaf angles from plant images procured using regular 6‐megapixel cameras. LAX is targeted at quantifying subtle changes in leaf angles in individual plants measured at densely spaced time points throughout development to identify spatial and temporal patterns of changes that occur at a shorter timescale of hours and days. The authors quantified leaf angle changes from multiple leaves in individual maize and sorghum plants subjected to water deprivation. LAX leaf angle measurements were able to reveal leaf angle changes coinciding with leaf wilting down to an interval of one minute in each plant. This high resolution provides spatiotemporal information, differentiating individual leaves in the plants that showed wilting from those that did not at any given time point.

One challenge inherent in high spatiotemporal information is how best to quantify differences in patterns of change over time so that informative comparisons can be made between distinct genotypes. Germination is conventionally scored as the percentage of seeds germinated at a single time point after planting. However, particularly for wild species that exhibit seed dormancy, germination can be a complex process with different timing of germination among individuals within genetically uniform populations. While a range of approaches have been proposed to summarize the complex behavior of the germination of a population of seeds, Talská et al. (2020) propose an approach based on smoothing splines and functional regression. Their approach is sensitive to both the proportions of seeds that ultimately germinate and the timing of germination across the window of observation. The authors evaluate their method against previously proposed approaches to quantifying germination behavior using simulated data and experimental data from a set of 105 wild pea accessions.

Another challenge of advanced data acquisition techniques is the deluge of data, often collinear, resulting in more predictors than observations. This challenge is particularly true with hyperspectral data, as reflection at hundreds of wavelengths can be measured on a single sample, necessitating the use of data reduction techniques. However, the potential advantages of hyperspectral imaging outweigh the challenges, as reflectance at wavelengths across different parts of the spectrum lets us “see” chemical changes in plants not yet visible to the naked eye or even standard imaging techniques. Ugarte Fajardo et al. (2020) apply partial least squares–penalized logistic regression and hyperspectral biplot techniques to the biological problem of early detection of black leaf streak disease in banana. Their methods allow for highly accurate and sensitive identification of the disease while still in the presymptomatic phase, before devastating spread and irreversible damage are caused by the fungus.

In the coming years, it seems likely that integrating new high‐throughput phenotyping methodologies to address questions relevant to plant genetics, physiology, and biochemistry will be commonplace. Nepal et al. (2020) demonstrate how a range of new phenotyping approaches developed over the past several years can be employed to address questions of gene‐by‐gene interactions. Nepal and colleagues employed a commercial imaging system to track the growth of plants under control, drought, and stress conditions, and a second technology—based on spectral analysis—that allowed rapid measurements of photosynthetic parameters. Their study employed these technologies to study the interactions of two genes, *MIOX4*—a *myo*‐inositol oxygenase, the first enzyme in the pathway for synthesizing ascorbate in plants—and *AVP1*, which is a pyrophosphate‐energized vacuolar membrane proton pump. The authors found that a combined overexpression line accumulates more biomass than wild type or single gene overexpression lines under drought conditions. They also show that increases in both linear electron flow and photosystem II efficiency accurately predict observed increases in seed count at maturity.

The collection of papers in this special issue represents work extending multiple scales in both biological organization and data analysis. At the biological scale, these papers encompass field‐level traits, traits among individual plants, and gene–gene interactions within a plant. Similarly, from a data analysis perspective, articles in this issue cover aspects including data acquisition and processing, algorithms for data analysis, applications in high‐throughput phenotyping for early detection of stress, and methods for differentiating growth and stress tolerance levels in gene‐by‐gene interaction studies. It seems quite likely that in coming years, just as the tools of molecular biology and, later, bioinformatics originated first in specialized disciplines, many of the phenotyping tools and analytical approaches reflected here will become standard parts of the plant biology toolkit deployed as needed to address questions of plant biology, genetics, and breeding.

## Author Contributions

S.K.K.R. Conceptualization (equal); Writing ‐ Original Draft (equal); Writing ‐ Review & Editing (equal). A.M.T. Writing ‐ Original Draft (equal); Writing ‐ Review & Editing (equal). J.C.S. Writing ‐ Original Draft (equal); Writing ‐ Review & Editing (equal).
